# Stripping Voltammetric Assay of Trace Technetium with a TOPO Coated Glassy Carbon Electrode

**DOI:** 10.6028/jres.093.132

**Published:** 1988-06-01

**Authors:** J. M. Torres Llosa, H. Ruf, K. Schorb, H. J. Ache

**Affiliations:** Kernforschungszentrum Karlsruhe, Institut für Radiochemie, D-7500 Karlsruhe, Federal Republic of Germany

It has been the goal pursued in the investigations described here to determine technetium trace concentrations using the sensitive method of stripping voltammetry with a chemically modified working electrode which responds in a highly selective manner. The attention was focused on the most stable + 7 oxidation state of the radioelement, which is present in the majority of cases while searching also for the possibility of discriminating Tc-IV which sometimes occurs simultaneously in the samples to be analyzed.

One kind of electrode modification which was considered promising in this respect consisted of coating a glassy carbon electrode (GCE) with trin-octylphosphinoxide (TOPO) which already proved its worth in stripping voltammetric uranium assays of sea water samples without requiring an additional separation step [1,2]. For it is known that, similar to uranium, technetium also reacts with TOPO to give a stable complex [3] which can be extracted from hydrochloric acid solutions and the composition of which is reported to be HTcO_4_·2 (TOPO) [4].

According to this consideration it should be possible to preconcentrate technetium from chloride-containing acid solutions on the surface of a TOPO coated GCE (TOPO-GCE) via complexation. This approach in principle should be capable of exploitation for stripping voltammetric technetium assays as in the case of uranium.

Since the coefficient of Tc-VII distribution in the TOPO-HCI system attains its maximum of slightly over 100 at about 3 *M* HCI [3], which is equivalent to an extraction extent of at least 99%, it was obvious to attempt enrichment from about 3 *M* HCI acid solutions.

It became apparent that the expected accumulation of technetium from strongly HCl-acid solutions according to the outlined principle actually takes place [5]. (The technetium isotope used in this work was the long-lived Tc-99.) A glassy carbon electrode modified with TOPO results in a well defined reduction peak after contact of only some minutes duration with solutions of low pertechnetate concentrations down to the 10^−8^ *M* level. The potential of the signal which occurs as a peak during measurement in the DP mode is approximately −350 mV (vs Ag/AgCl). The signal is generated after enrichment if a voltage scan starting at 0 V is directed towards negative potentials. Stripping voltammograms recorded in this way for Tc-VII solutions of different concentrations are shown in figure 1. It is obvious from the correlation coefficient *R* =0.9987 calculated from the measured signal heights and the amounts of technetium present that a linear relationship exists which is well suited for calibration purposes.

As a confirmation of the enrichment effect the analytical technetium signal rises first at a linear rate with longer enrichment time (see fig. 2). But if the reaction time is extended more this increase in signal amplitude becomes visibly smaller which is evidence of the limitation of the active electrode surface capacity.

It appears from figure 3 that it is actually the TOPO layer (prepared by pipetting and heat drying an appropriate volume of an ethanolic TOPO solution on the electrode) which is responsible for technetium fixation. In the absence of TOPO no enrichment at all takes place at the open-circuit GCE.

Best sensitivities are attained if high negative pulse amplitudes (−100 mV or more) as well as a voltage scan of approximately 60 mV·s^−1^ are employed.

Which electrochemical reaction of Tc-VII underlies the analytical signal recorded has not yet been investigated. Statements which have been made in the literature with respect to polarographic waves at similar half-wave potentials recorded so far by mercury electrodes in acid pertechnetate solutions are contradictory. This equally applies to measurements performed in 4 *M* HCI.

In accordance with the reported possibility of extracting Tc-IV with TOPO [6], Tc-IV can also be enriched at the TOPO-GCE and subsequently a voltammetric peak can likewise be observed. However, the potential of this peak unfortunately deviates only slightly from that of Tc-VII so that perfect assay of both species in this way is difficult. In order to elucidate the extent of interference, measurements were made with Tc-IV (prepared by electrolytic reduction of Tc-VII in 6 *M* HCI according to [7]) to which various amounts of Tc-VII had been added. The stripping voltammograms thereby obtained in favorable cases seem to enable a rough estimate to be made of the Tc-IV and Tc-VII fractions.

Uranium, as reported [1,2], gives rise to a stripping peak which is situated at −480 mV (vs. Ag/AgCl) under the experimental conditions chosen in this work. While the obstacles resulting from low uranium content should not be serious, samples containing a preponderance of uranium preclude the assay of technetium with the TOPO-GCE.

Except for some other metals which can be extracted from strongly acid solutions using TOPO (this applies to Fe, Mo, Zn, Nb and Sn-IV [3]) as well as for considerably interfering oxygen, the technetium assay studied here is hardly disturbed by other elements.

The analytical procedure described will be profitable not the least to the present relevant investigation of solutions which arise from the prophylactic studies on the leachability of technetium after the attack of chloride brines in vitrified nuclear waste proposed for deep disposal in salt mines.

Also stripping chronopotentiometric measurements performed at the technetium loaded TOPO-GCE will provide analytically interesting data. Pronounced potential-time plots are recorded during galvanostatic reduction both with Tc-VII and Tc-IV; the transition times observed are dependent on the technetium concentration.

## Figures and Tables

**Figure 1:**
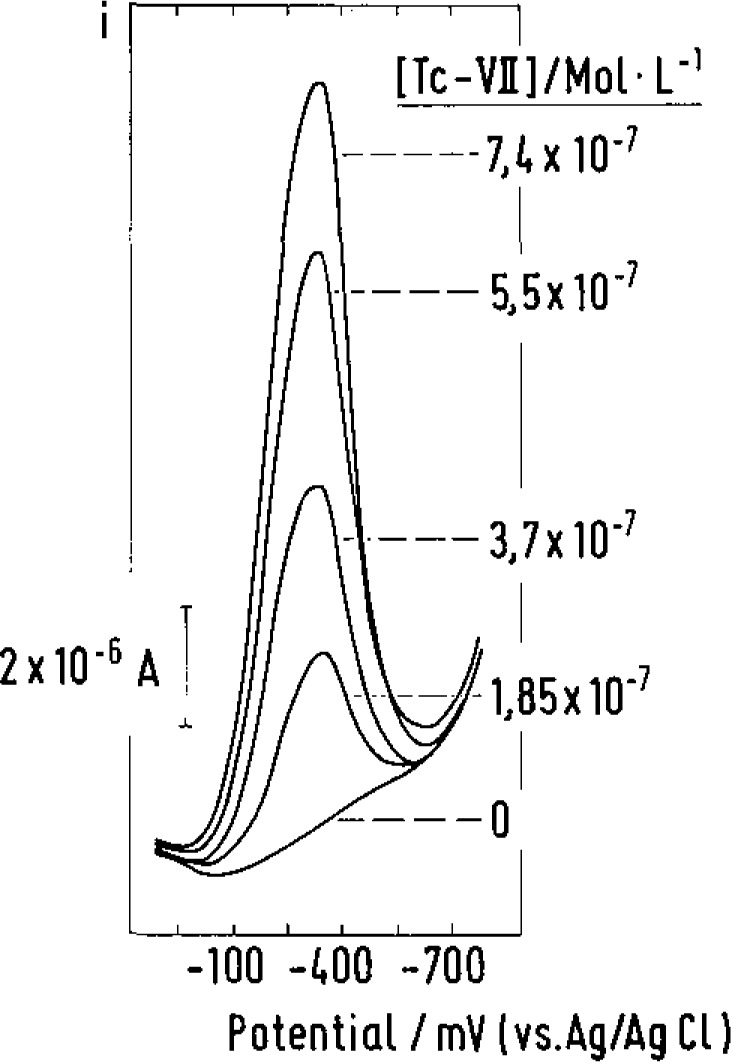
DP stripping voltammograms recorded in TcO_4_^−^ solutions of different concentrations with the TOPO-GCE after enrichment in the absence of an applied electrolytic voltage. Supporting electrolyte: 3 *M* HCI; enrichment time: 200 s; pulse amplitude: −250 mV; scan rate: 10 mV·s^−1^.

**Figure 2:**
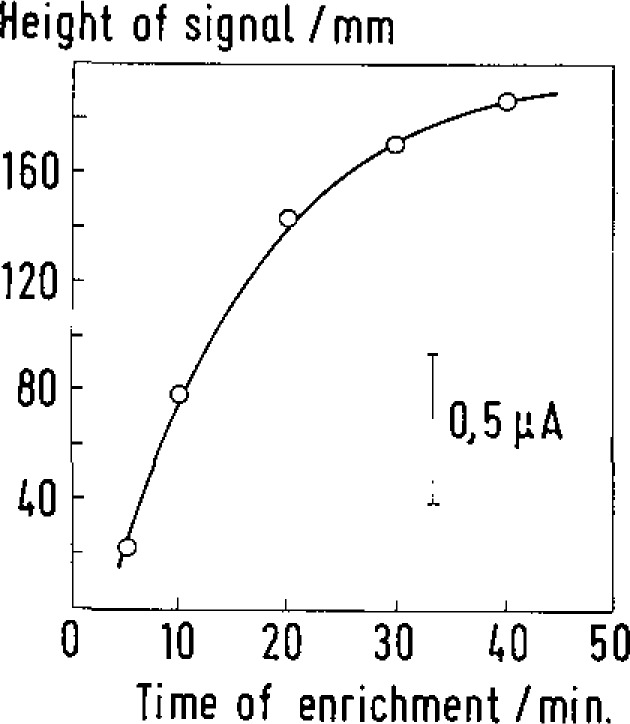
DP stripping voltammograms recorded with the TOPO-GCE in a 10^−7^ *M* TcO_4_^−^ solution after different enrichment times. Pulse amplitude: −100 mV; scan rate: 15 mV·s^−1^.

**Figure 3:**
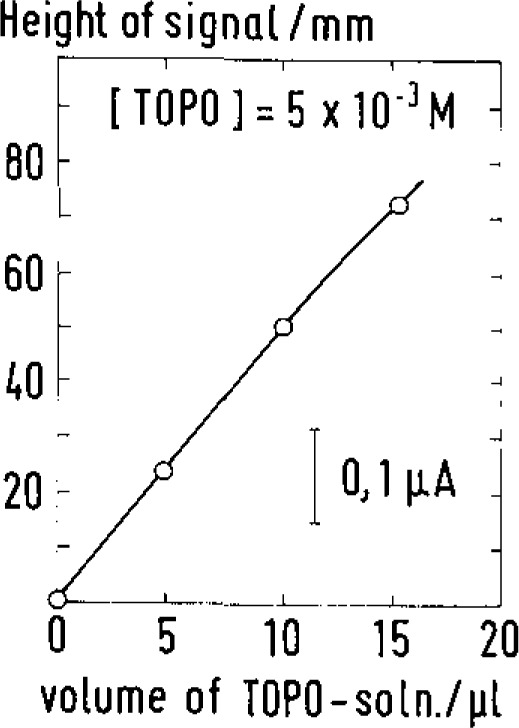
DP stripping voltammetric Tc signal heights at −350 mV (versus Ag/AgCl) recorded at the TOPO-GCE as a function of enrichment time. Tc-VII concentration: 10^−7^ *M*; pulse amplitude: −100 mV; scan rate: 15 mV·s^−1^.
